# Impact of the COVID-19 Lockdown on the Body Composition and Physical Performance of Athletes: A Systematic Review with Meta-Analysis and Meta-Regression

**DOI:** 10.3390/healthcare11162319

**Published:** 2023-08-17

**Authors:** Bruno Viana Rosa, Alberto Jimenez Maldonado, Ayrton Oliveira de Araújo, Lucas Melo Neves, Fabricio Eduardo Rossi

**Affiliations:** 1 Immunometabolism of Skeletal Muscle and Exercise Research Group, Department of Physical Education, São Paulo State University, Presidente Prudente 19060-900, SP, Brazil; brunovianarosa@gmail.com (B.V.R.); ayrtinaraujo@gmail.com (A.O.d.A.); 2 Facultad de Deportes Campus Ensenada, Universidad Autónoma de Baja California México, Mexicali 22890, BC, Mexico; jimenez.alberto86@uabc.edu.mx; 3 Post-Graduate Program in Health Sciences, Santo Amaro University, Sao Paulo 04829-300, SP, Brazil; lucasmeloneves@uol.com.br; 4 Bipolar Disorder Program (PROMAN), Department of Psychiatry, Medical School, University of São Paulo, Pacaembu 05508-220, SP, Brazil; 5 Graduate Program in Science and Health, Federal University of Piauí (UFPI), Teresina 64049-550, PI, Brazil; 6 Graduate Program in Movement Science, São Paulo State University, Presidente Prudente 19060-900, SP, Brazil

**Keywords:** athletes, physical performance, lockdown, body composition

## Abstract

Sporting events were cancelled, and sports training was banned to prevent the spread of COVID-19. These changes during the COVID-19 pandemic decreased the physical activity levels, increased sedentary time, and also impaired the mental health of elite and sub-elite athletes. The impact on body composition and physical performance is not clear, however, especially considering a systematic review with meta-analysis. Thus, our objective was to conduct a review in accordance with the PRISMA Statement studies published in scientific journals (PubMed, Web of Science, or Scopus databases) that investigated the effect that social distancing during the COVID-19 pandemic had on the physical performance (muscle power, cardiorespiratory capacity, and sprint) or body composition (body weight, percentage of fat, fat mass, and fat-free mass) of athletes. Data from 24 studies indicate that, throughout the global lockdown, the athletes maintained muscle power, cardiorespiratory capacity, and sprint, and prevented significant changes in fat mass and fat-free mass. However, the total body weight (meta-analysis with 18 studies), showed a significant increase (*p* = 0.006), with a small ES = 0.12; 95% CI = 0.04 to 0.21. Furthermore, the time of follow-up, level of training, and the age of the athletes were possible moderators of these effects. The data reinforce the importance of general strength and endurance exercises sessions to maintain physical fitness during non-competitive periods or due to the mandatory lockdown.

## 1. Introduction

The COVID-19 pandemic was caused by SARS-CoV-2 (severe acute respiratory syndrome coronavirus-2). In March 2020, the World Health Organization (WHO) published guidelines to prevent the spread, due to a high rate and number of progressing infections, which involved mainly social restriction; therefore, sports events were canceled, and sports training was banned [1]. A recent systematic review conducted by Jurecka et al. [2] demonstrated that athletes decreased their physical activity levels, increased sedentary time, and experienced impaired mental health during the COVID-19 pandemic. The authors suggested that the temporary restriction was associated with a decrease in overall physical fitness and the number of days and hours of training.

Likewise, other researchers identified that, during the social distancing period, the athletes decreased their total volume of training; however, most of them tried to maintain cardiorespiratory fitness and muscular strength, rather than exploiting sports-specific training [3]. These measures were taken to cope with the need to maintain cardiorespiratory fitness, velocity, power, and morphological functions, relevant factors to achieve successful results among highly trained athletes, independently of their sport. Furthermore, athletic performance improvement is often associated with a reduction in fat mass (FM) and increased fat-free mass (FFM) [4]. In accordance with this, Siders et al. found a positive relationship between sprint swimming performance and percentage of fat mass and a negative relationship with FFM in women [5]. Furthermore, athletes with lower FM showed higher aerobic capacity and more biochemical blood markers associated with an anabolic process, which may considerably affect the exercise capacity of athletes [6]. However, only 4 weeks of detraining is enough to reduce 6% to 20% of maximal oxygen uptake ($\overset{˙}{V}$O2peak) in highly trained athletes [7].

Spyrou et al. found that a short period of lockdown (9 weeks) decreased the sprint, countermovement jump (CMJ), rate of force development, peak power, velocity, and landing peak force of elite futsal players [8]. On the other hand, Fikenzer et al. and Grazioli et al. did not verify a significant difference in cardiorespiratory fitness after 8 weeks in handball and soccer players, respectively, although higher fat mass was found for the soccer players in Grazioli’s study [9,10]. Despite differing statements recommending the safe return to training and competition after the lockdown caused by the COVID-19 pandemic [11,12], the retraining period effect on these variables is not yet clear, particularly after a long detraining season. We highlight the study of Silva et al., which investigated the impact of long-period detraining due to the COVID-19 social restrictions (8 months and 1 year) on young badminton athletes. The authors showed that the athletes who stopped daily training routines for 1 year due to the COVID-19 social restrictions presented higher FM and lower FFM than the athletes who returned to regular daily training 4 months earlier; however, no significant differences were observed for $\overset{˙}{V}$O2peak and handgrip strength [13].

In regard to the previous information, it is possible to identify that there is no consensus about the effects of the COVID-19 pandemic on the physical performance and body composition of athletes; therefore, a clear understanding of the impact of COVID-19 on the physical fitness of athletes is essential to inform coaches, sports physiologists, and athletes when making decisions concerning the initial load and progressions during the return to training and matches. Furthermore, safe and healthy strategies can be developed to mitigate physiological and morphological responses for athletes to return to competition-level readiness. 

For athletes, it is relatively easy to suffer a loss in body mass and FM due to higher energy expenditure during exhausting training routines and competition, however, it is hard for them to maintain the FFM required when performing muscular work [14]. On the other hand, long periods of discontinuing training might induce body mass and FM gain, decrease FFM, and impair performance throughout the season. 

Thus, this is the first study to use both a meta-analysis and meta-regression to investigate the impact of the COVID-19 pandemic on the body composition (fat mass and fat-free mass) and physical fitness (muscle power, cardiorespiratory capacity, and sprint) of athletes, as well as to explore the possible moderators of these effects, such as the immediate impact of the initial lockdowns in 2020 and long periods of detraining caused by the COVID-19 social restrictions. Additionally, we investigated the potential influence that the level of training and age had on the athletes, since elite and non-elite athletes could show different morphological and physical responses during this period and across their life. We hypothesized that the COVID-19 pandemic would negatively affect the body composition, with higher fat mass and lower FFM, and would decrease cardiorespiratory fitness and muscle power. 

## 2. Materials and Methods

This review has been reported in accordance with the PRISMA Statement (preferred reporting items for systematic reviews and meta-analyses) checklist [15].

### 2.1. Search Strategy

Searches were performed in PubMed, Web of Science, and Scopus using the following word combinations: (“COVID-19” OR “SARS-CoV-2” OR coronavirus OR pandemic OR “social isolation” OR quarantine OR epidemic) AND (Athletes OR “high-trained” OR players OR sportsmen OR “elite athletes” OR “amateur athletes” OR “young athletes”) AND (“Physical fitness” OR performance OR training OR exercise OR obesity OR “weight loss” OR body composition OR anthropometric OR “adipose tissue” OR adiposity OR “fat mass” OR “visceral fat” OR “lean body mass” OR “muscle mass” OR “fat free mass”). 

The filter was applied to studies published from 2020 onwards, given that the COVID-19 infection only spread around the world in early 2020, and the confinements caused by the virus only started in February and March in most countries, according to the WHO [1]. 

### 2.2. Eligibility Criteria

Prospective and retrospective cohort articles, interrupted time series, case series, and clinical trials published in scientific journals, indexed in databases (PubMed, Web of Science, or Scopus), that investigated the effect social distancing during the COVID-19 pandemic on the physical performance or body composition of athletes were included. Articles were required to contain data before and after social distancing, or some period during the pandemic, in elite or sub-elite athletes (for the first population, we considered elite athletes who had competed in international or national sports events; whereas sub-elite athletes were considered those that competed regionally or recreationally). Athletes were required to be engaged in regular training during the week, with participation in national or international competitions. The exclusion criteria included articles with self-reported performance or composition and studies that included athletes with some type of disability (i.e., palsy or other diseases); moreover, athletes younger than 1 year old were not included, as well as studies that compared athletes who had COVID-19. Articles published in the form of abstracts, dissertations, and theses were not considered for this review.

### 2.3. Selection Process

The entire study selection process, from titles, abstracts, and full text, was carried out by two independent reviewers (BVR and AOA). In cases of unresolved conflict, a third reviewer would help to decide (FER). The process of removing duplicates and selecting studies was performed using the Rayyan web application [16].

### 2.4. Data Collection Process

The data of interest were taken from the articles by 3 authors (BRV, AOA, and FER), where each author had an average of 20 articles for data extraction. Data were collected manually from studies and compiled in excel sheets. Studies or data not found were requested by email to the authors. 

### 2.5. Data Items

For the pre- and post-confinement physical fitness outcomes, the following topics and data were extracted from each study: author and year, sport, level of training, number of subjects, gender, age, weeks of follow-up, performance or body composition measurement evaluation. 

Additionally, the study design and training protocol during confinement were extracted (see Supplementary Materials).

Muscle power measured by counter movement jump (CMJ), cardiorespiratory fitness measured by $\overset{˙}{V}$O2peak (direct or indirect tests), and sprint (5 m, 10 m, and 30 m tests) were used for the meta-analysis of performance. Additionally, body mass (kg), percentage of fat mass (%), fat mass (kg), and fat-free mass (kg) were used for the meta-analysis of body composition.

In studies that presented data collection at different moments in time, data were used only from the moment closest to pre-pandemic (before COVID-19 pandemic) and closest to post-pandemic social restriction.

### 2.6. Study Risk of Bias Assessment

Two independent reviewers (BVR and FER) assessed the quality of the studies. Methodological quality was not an inclusion criterion. The risk of bias was assessed using the Joanna Briggs Institute (JBI) critical appraisal tool [17]. Each item was assigned a high, low, or unclear risk of material bias, and the quality of all studies was analyzed.

### 2.7. Data Synthesis

A random-effects model was used in all analyses, owing to an expectation of heterogeneity of data across studies. Standardized mean differences (SMD) in body composition and performance variables from pre- to post-COVID-19 pandemic social distancing were utilized to calculate the effect size (ES). We calculated the SMD for individual studies and then pooled the data using a random-effects meta-analysis. SMD was estimated from pre- to post-pandemic, with 0.7 correlations. The interpretation of the magnitude of standardized mean difference was as follows: 0 to <0.30 | small, | >0.30 | to | <0.8 | moderate, and | >0.80 | large [18].

Heterogeneity among studies was verified with the I^2^ statistic, with thresholds set at I^2^ = 25% (low), I^2^ = 50% (moderate), and I^2^ = 75% (high) [18,19,20,21]. Computations were carried out using Comprehensive Meta-Analysis Version 4 (Biostat Inc., Englewood, NJ, USA) [22].

We also performed sensitivity analyses to assess whether comparable effects were still observed following the removal of low-quality information (≤5 points on the JBI critical appraisal tool).

Meta-regression analysis of variables with ten or more studies was carried out. The moderators used were as follows: (a) age—years; (b) follow-up time—weeks; and (c) level of training—elite or non-elite. The significance level adopted was *p* ≤ 0.05.

## 3. Results

**Figure 1:**
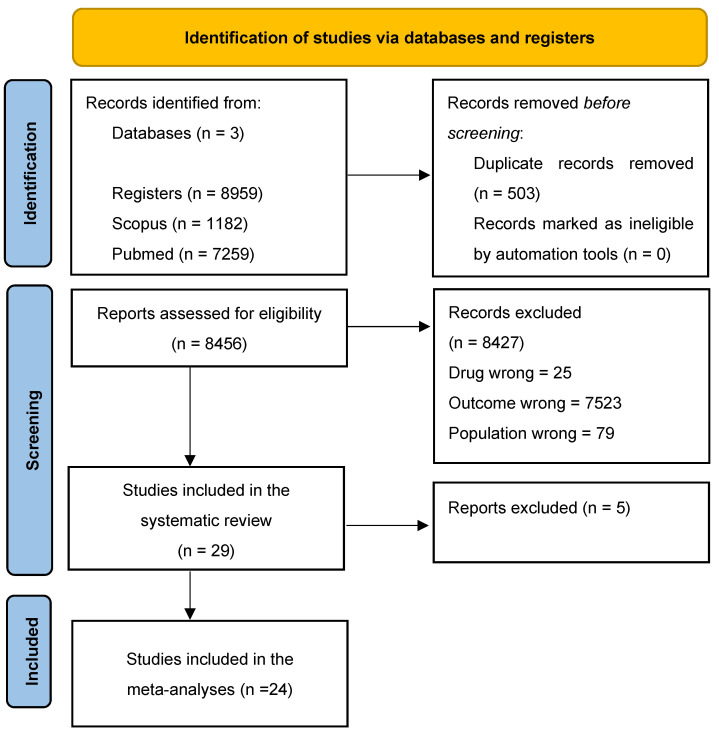
The flowchart of study selection.

The initial search identified 8959 potentially relevant articles. After the removal of duplicates (503 studies), 8456 records remained. Of these, we removed 8427 studies based on their title and abstract (Drug wrong = 25; Outcome wrong = 7523; Population wrong = 79; Publication type = 62; Study design = 735; and Study duration = 3), and 29 studies were included in our systematic review. However, some articles did not provide pre- or post-pandemic data as mean and standard deviation (even after we requested them by email from the corresponding author), therefore, the meta-analysis considered 24 studies. The study selection process is detailed in Figure 1.

### 3.1. Characteristics of Included Trials and Study Quality

The 29 studies selected for systematic review [8,9,10,13,23,24,25,26,27,28,29,30,31,32,33,34,35,36,37,38,39,40,41,42,43,44,45,46,47] and the quality of all of the studies analyzed are presented in Table 1. The details of these studies are presented in Table 2. In summary, a total of 691 athletes were included from 23 studies with men (593 athletes), 2 studies with women (32 athletes), and 4 studies with men and women (66 athletes). In relation to modalities, nine different modalities were identified (badminton = 2, basketball = 1, cycling = 1, fencing = 2, handball = 2, indoor soccer = 1, kickboxing = 1, soccer = 18, and combat Sports = 1). The variables identified were body mass = 21, fat-free mass = 8, fat mass = 4, fat percentage = 10, CMJ = 14, and $\overset{˙}{V}$O2peak = 10. 

### 3.2. Meta-Analysis

**Figure 2:**
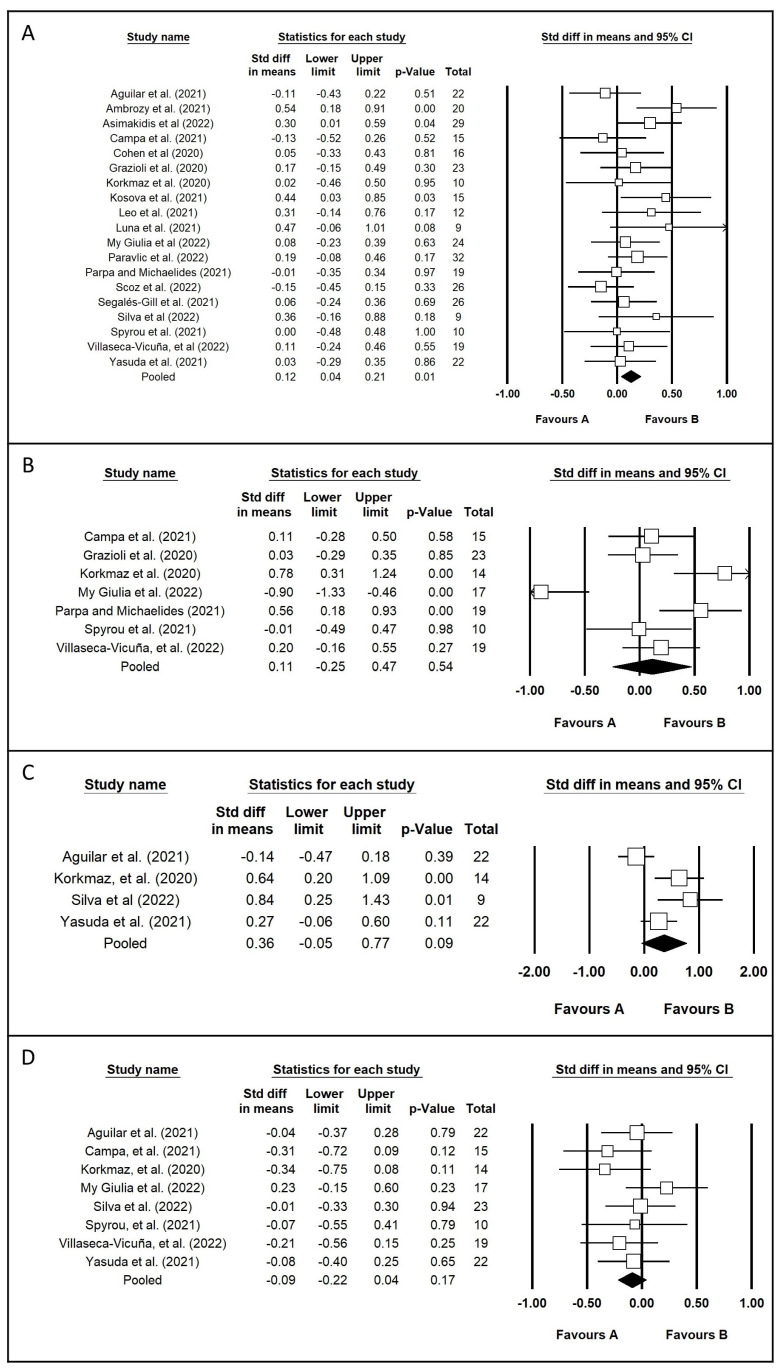
Forest plot of body composition variables: (**A**) = Body mass [8,10,13,23,25,26,27,28,32,33,34,35,36,37,38,41,42,44,47]; (**B**) = Percentage of fat [8,10,27,32,36,38,44]; (**C**) = Fat mass [13,23,32,47]; (**D**) = Fat-free mass [8,13,23,27,32,36,44,47]. Favor A = Decrease value of Variable; Favor B = Increase value of Variable.

Of the 29 studies considered for the systematic review, 5 studies [24,31,43,45,46] were excluded. In summary, the motivation of exclusion was as follows: the number of subjects per group was not clear [24]; the data of $\overset{˙}{V}$O2peak was reported in other measurement units (meters) [31]; and no access to data in mean and standard deviation (even after email request) [43,45,46]. A total of 24 studies [8,9,10,13,23,25,26,27,28,29,30,32,33,34,35,36,37,38,39,40,41,42,44,47] and 447 athletes were included in this meta-analysis. This study resulted in six forest plots. In detail, we present (Figure 2) the following four forest plots of body composition:

Body mass variable [8,10,13,23,25,26,27,28,32,33,34,35,36,37,38,41,42,44,47]. 

Percentage of fat variable [8,10,27,32,36,38,44].

Fat mass variable [13,23,32,47].

Fat-free mass variable [8,13,23,27,32,36,44,47].

In summary, body weight analysis, included 18 studies and 336 participants, with a range from 9 to 32 participants. The mean of changes was 1.1 kg (range from −1.1 to 4.5 kg), or 1.7% (range from −1.4 to 7.4%). We found a significant (*p* = 0.006) change (increase in body mass) (ES = 0.12; 95% CI = 0.04 to 0.21, Q = 20.403, with 18 degrees of freedom, and *p* = 0.01, I^2^ = 17%).

Percentage of fat analysis included seven studies and 117 participants, with a range from 10 to 23 participants. The mean of changes was 0.5% (range from −1.5 to 2.5%). We found a nonsignificant (*p* = 0.54) change (ES = 0.11; 95% CI = −0.25 to 0.47, Q = 34.655, with 6 degrees of freedom, and *p* < 0.001, I^2^ = 83%).

Fat mass analysis included four studies and 67 participants, with a range from 9 to 22 participants. The mean of changes was 1.3 kg (range from −0.4 to 2.8 kg), or 11.6% (range from −4.0 to 22.0%). We found a nonsignificant (*p* = 0.087) change (ES = 0.36; 95% CI = −0.05 to 0.77, Q = 12.440, with 3 degrees of freedom, and *p* < 0.006, I^2^ = 76%).

Finally, fat-free mass analysis, included eight studies and 142 participants, with a range from 10 to 23 participants. The mean of changes was −0.3 kg (range from −1.2 to 1.3 kg), or −0.9% (range from −3.3 to 1.7%). We found a no significant (*p* = 0.17) change (ES = −0.09; 95% CI = −0.22 to 0.04, Q 6.050, with 6 degrees of freedom, and *p* < 0.534, I^2^ = 0%).

**Figure 3:**
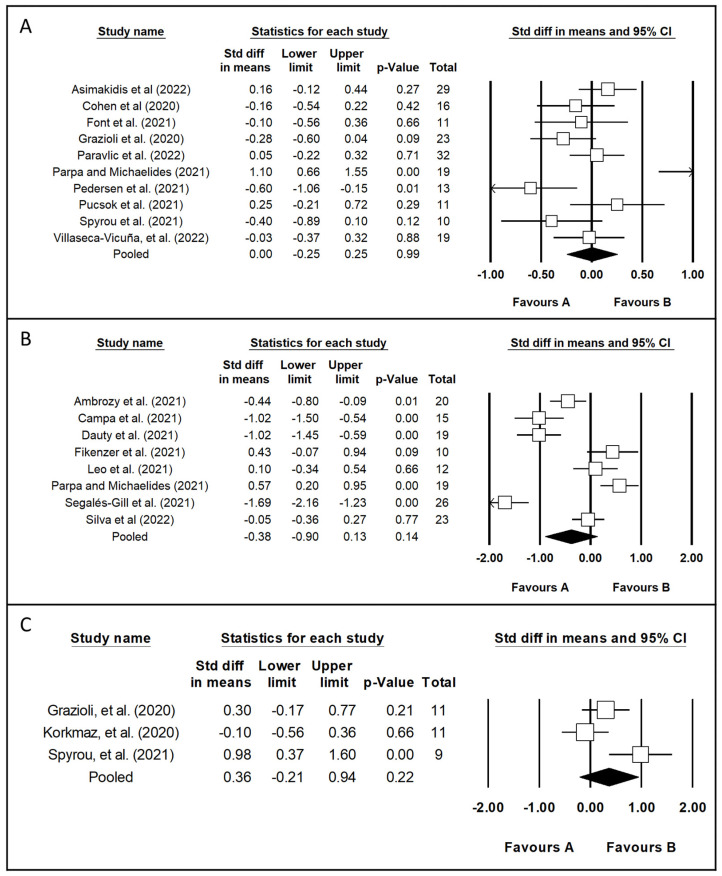
Forest plot of performance variables: (**A**) = Counter movement jump [8,10,26,28,30,37,38,39,40,44]; (**B**) = $\overset{˙}{V}$O2peak [9,13,25,27,29,34,38,42]; (**C**) = Sprint [8,10,22]. Favor A = Decrease value of Variable; Favor B = Increase value of Variable.

Additionally, we present in Figure 3 a total of three forest plots of performance variables, as follows:

CMJ test [8,10,26,28,30,37,38,39,40,44].

$\overset{˙}{V}$O2peak variable [9,13,25,27,29,34,38,42].

Sprint [8,10,22].

In summary, CMJ analysis, included 10 studies, with 183 participants, with a range from 10 to 32 participants. The mean of changes was 0.3 cm (range from −1.7 to 6.1 cm), or 0.8% (range from −4.7 to 15.4%). We found a nonsignificant (0.99) change (ES = 0.00; 95% CI = −0.25 to 0.25, Q 39.334, with 9 degrees of freedom, and *p* < 0.001, I^2^ = 77%).

$\overset{˙}{V}$O2peak analysis, included eight studies, with 144 participants, with a range from 10 to 26 participants. We found a nonsignificant (*p* = 0.14) change (ES = −0.38; 95% CI = −0.90 to 0.13, Q 88.842, with 7 degrees of freedom, and *p* < 0.001, I^2^ = 92%).

Finally, data of sprint included from three studies, with 31 participants, with a range from 9 to 11 participants. We found a nonsignificant (*p* = 0.22) change (ES = 0.36; 95% CI= −0.21 to 0.94, Q 146.729, with 2 degrees of freedom, and *p* < 0.001, I^2^ = 99%).

### 3.3. Sensibility Analysis

A sensibility analysis was performed excluding studies with low quality (≤5 points on the JBI critical appraisal tool—see Table 1). There was a total of 13 studies (n = 348) that were included in this analysis [8,9,10,13,23,24,27,29,31,36,37,38,41].

In relation to the body composition variables, we did not find significant ES—Body mass variable (nine studies, n = 180) [8,10,13,23,27,36,37,38,41], the effect size was SMD = 0.01 (95% CI = −0.09–0.10, *p* = 0.91), and heterogeneity I^2^ = 95% (Q = 166.75, *p* < 0.00); Percentage of fat variable (five studies, n = 82) [8,10,27,36,38], the effect size was SMD = 0.03 (95% CI = −0.31–0.39 *p* = 0.828), and heterogeneity I^2^ = 99% (Q = 490.55, *p* = 0.00); Fat mass variable (two studies, n = 31) [13,23], the effect size was SMD = 0.11 (95% CI = −0.38–0.60, *p* = 0.663), and heterogeneity I^2^ = 99% (Q = 77.41, *p* < 0.00); Fat-free mass variable (five studies, n = 84) [8,13,23,27,36], the effect size was SMD = −0.01 (95% CI = −0.01–0.01, *p* = 0.718), and heterogeneity I^2^ = 86% (Q = 28.26, *p* < 0.00).

As for the body composition variables, in relation to the performance variables, we did not find significant ES–CMJ test (four studies, n = 84) [8,10,37,38], the effect size was SMD = 0.12 (95% CI = −0.45–0.69, *p* = 0.69), and heterogeneity I^2^ = 90% (Q = 28.97, *p* < 0.00); $\overset{˙}{V}$O2peak variable (five studies, n = 86) [9,10,13,27,29,38], the effect size was SMD = −0.21 (95% CI = −0.85–0.43, *p* = 0.52), and heterogeneity I^2^ = 92% (Q = 47.94, *p* < 0.00). Due to the low number of studies on the sprint variable, sub-analyses/sensibility analysis were not performed.

### 3.4. Meta-Regression 

Table 3 presents the meta-regression of moderators for body weight and CMJ variables. Considering body weight, a significant moderator was observed in age—years (β = −0.023, 95% CI = −0.027 to −0.020, *p* < 0.001), follow-up time—weeks (β = −0.006, 95% CI = −0.005 to −0.008, *p* < 0.001), and level—elite or non-elite (β = −0.169, 95% CI = −0.200 to −0.128, *p* < 0.001). Considering the CMJ, a significant moderator was observed in age (β = −0.008, 95% CI = −0.009 to −0.006, *p* < 0.001), follow-up time (weeks) (β = −0.065, 95% CI = −0.074 to −0.057, *p* < 0.001), and level (elite or non-elite) (β = 0.076, 95% CI = 0.017 to 0.135, *p* < 0.001). 

## 4. Discussion

The current systematic review and meta-analysis revealed that athletes maintained their physical performance and prevented fat mass gain and fat-free mass loss during the COVID-19 pandemic; however, they gained total body mass. This main finding is important, since most of them trained alone (80%) and intended to maintain their physical capacity, with a focus on body mass (65%) and cardiovascular (59%) capacities, while sports events and regular training routines were banned, and less than the 40% of athletes worldwide were able to maintain their sport-specific training [3].

Christensen et al. [48] demonstrated that a high-intensity training program is a feasible intervention to maintain physical fitness after two weeks in football players; however, the participants who interrupted their training completely showed a severe reduction in their physical fitness. Our meta-analysis demonstrated that the COVID-19 pandemic did not negatively affect the fitness level. However, it is important to mention that, during this period, most of the athletes that were analyzed performed home-based training programs. Our results support the recommendations previously mentioned by other authors [12]; specifically, the authors emphasized that the regular practice of exercise training at home during the social distancing period was the best way to attenuate the loss of function in athletes. However, during the regular season, athletes need to focus both on physical fitness and technical-tactical skills. On the other hand, during the COVID-19 pandemic, their training activities were mainly focused on physical conditioning, which, in turn, probably induced higher positive adaptations.

Mujika and Padilla [7] found a reduction of 6% to 20% $\overset{˙}{V}$O2max after a 4-week detraining period in highly trained athletes. However, the findings obtained in our work showed that home-based training was a practicable intervention to maintain cardiorespiratory fitness during lockdown. Nevertheless, it was noticeable that there were only eight studies verifying this variable; furthermore, by using different ergometers or tests, such as a treadmill and shuttle-run test, the heterogeneity in the procedures can be a risk of bias in the results obtained [49]. Therefore, caution should be taken when interpreting that data, and further studies should be conducted to confirm our findings. 

The results shown in Figure 3 indicate that the muscle power and sprint were not significantly affected by the COVID-19 lockdown. Koundourakis et al. verified a decrease in SJ and CMJ performance after six weeks of detraining in both elite and sub-elite soccer players [50]; however, no differences were identified in recreationally strength-trained men [49]. The data suggested that athletes with high initial strength levels may suffer a reduction in power, while the jump ability of individuals with lower strength levels may not be influenced by a period of inactivity. The I^2^ value for those variables was high, indicating a considerable heterogeneity; therefore, we did not disregard the fact that the participants analyzed in this meta-analysis had different levels of lower-body strength (in accordance with their sports demands), and this situation could be a partial explanation for the null statistical effects found. 

In addition, a detraining period can reduce the fast-twitch fibers that are associated with maximal strength, explosive power, and velocity [51], while a retraining period may induce supercompensation, which also can be a reason to maintain FFM. Chronic muscle contraction induces a variety of metabolic and morphological adaptations in contracted skeletal muscles to maintain homeostasis and minimize FFM loss, thus, the sufficient stimulation performed by athletes with greater volume and frequency attenuated FFM and would become sensitive again after a short detraining, or non-training, period [52].

Regarding body fat, there were no significant changes in fat mass or percentage of fat, although athletes increased their total body mass. In non-athletes, Javadi Arjmand et al. conducted a cohort study with 24,968 participants and found that high psychological distress was strongly associated with higher levels of emotional eating and high-sugar food intake [53]. In fact, the consumption of high caloric foods, due to impulse or anxiety, as well as lower levels of healthy eating habits were found, mainly at the start of the pandemic, which can maybe explain the changes in total body mass. This has also been a challenge for athletes during the home confinement caused by the COVID-19 pandemic [54]. However, further studies should be conducted on athletes to verify this hypothesis.

Furthermore, it is worth mentioning that most of the studies analyzed here were conducted on men. Concretely, from the 29 studies studied in the current meta-analysis, only 5 works involved women (2 of which were exclusive to women). Despite the controversy regarding the difference between the genders on physical performance, Ivey et al. reported that men retain the benefits of exercise training on muscle volume (despite 31 weeks of detraining), whereas women did not show the same ability [55]. On other hand, another study reported that women did retain the strength gained (as consequence of 9 weeks of strength training) after a period of 31 weeks, whereas, in men, the loss of muscle strength was significant after 31 weeks of detraining [56]. Those studies lead us to take care of the findings, mainly considering that gender is a variable that may regulate muscle quality (mass and muscle strength) in a detraining intervention. Therefore, future studies are needed in order to achieve a clear understanding about the gender effect on the physical performance of athletes during social distancing (i.e., the COVID-19 lockdown).

## 5. Conclusions

In summary, the athletes maintained muscle power, cardiorespiratory capacity, and sprint velocity, and prevented significant changes in their fat mass and fat-free mass during the COVID-19 pandemic; however, they increased their total body mass. Furthermore, the time of follow-up, the level of training, and the age of the athletes were possible moderators of these effects. Therefore, these data reinforce the importance of general strength and endurance exercise sessions to maintain physical fitness during non-competitive periods, or due to a mandatory lockdown. However, strategies such as combining training with diet to prevent body fat gain should be used, mainly if a long period of detraining is necessary. 

## Figures and Tables

**Table 1:** Risk of bias assessment assessed using the Joanna Briggs Institute (JBI) critical appraisal tool.

Author and Year	1	2	3	4	5	6	7	8
Aguilar et al. (2021) [23]	✓	✓	✓	✓	X	X	✓	✓
Alvurd et al. (2022) [24]	✓	✓	✓	✓	X	X	✓	✓
Ambrozy et al. (2021) [25]	X	✓	✓	✓	X	X	✓	✓
Asimakidis et al. (2022) [26]	X	✓	✓	✓	X	X	✓	✓
Campa et al. (2021) [27]	✓	✓	✓	✓	X	X	✓	✓
Cohen et al. (2020) [28]	X	✓	✓	✓	X	X	✓	✓
Dauty et al. (2021) [29]	✓	✓	✓	✓	X	X	✓	✓
Fikenzer et al. (2021) [9]	✓	✓	✓	✓	X	X	✓	✓
Font et al. (2021) [30]	X	✓	✓	✓	X	X	✓	✓
Freire et al. (2020) [43]	X	✓	✓	✓	X	X	✓	✓
Grazioli et al. (2020) [10]	✓	✓	✓	✓	X	X	✓	✓
Junaidi et al. (2021) [46]	X	✓	✓	✓	X	X	X	✓
Kalinowski et al. (2021) [31]	✓	✓	✓	✓	X	X	✓	✓
Korkmaz et al. (2020) [22]	X	X	✓	✓	X	X	✓	✓
Kosova et al. (2021) [33]	X	✓	✓	✓	X	X	X	✓
Leo et al. (2021) [34]	X	✓	✓	✓	X	X	✓	✓
Luna et al. (2021) [35]	X	✓	✓	✓	X	X	✓	✓
My Giulia et al. (2022) [36]	✓	✓	✓	✓	X	X	✓	✓
Paravlic et al. (2022) [37]	✓	✓	✓	✓	X	X	✓	✓
Parpa and Michaelides (2021) [38]	✓	✓	✓	✓	X	X	✓	✓
Pedersen et al. (2021) [39]	X	✓	✓	✓	X	X	✓	✓
Pucsok et al. (2021) [40]	X	✓	✓	✓	X	X	✓	✓
Scoz et al. (2022) [41]	✓	✓	✓	✓	X	X	✓	✓
Segalés-Gill et al. (2021) [42]	X	✓	✓	✓	X	X	✓	✓
Silva et al. (2022) [13]	✓	✓	✓	✓	✓	X	✓	✓
Spyrou et al. (2021) [8]	✓	✓	✓	✓	X	X	✓	✓
Valenzuela et al. (2021) [45]	X	✓	✓	✓	X	X	✓	✓
Villaseca-Vicuña et al. (2022) [44]	X	✓	✓	✓	X	X	✓	✓
Yasuda et al. (2021) [47]	X	✓	✓	✓	X	X	✓	✓

Caption: The Joanna Briggs Institute (JBI) critical appraisal tool with 8 items. 1—Were the criteria for inclusion in the sample clearly defined?; 2—Were the study subjects and the setting described in detail?; 3—Was the exposure measured in a valid and reliable way?; 4—Were objective, standard criteria used for measurement of the condition?; 5—Were confounding factors identified?; 6—Were strategies to deal with confounding factors stated?; 7—Were the outcomes measured in a valid and reliable way?; 8—Was appropriate statistical analysis used? ✓ = Yes; X = No.

**Table 2:** Characteristics of studies included in the systematic review.

Author and Year	Sport	Level of Training	N	Gender	Age	Weeks	BMI	Body Mass	Fat-Free Mass	Fat Mass	Fat %	CMJ	$\overset{˙}{V}$O2peak
Aguilar et al. (2021) [23]	Soccer	Elite	22	M	18 to 32	12		X	X	X			
Alvurd et al. (2022) [24]	Soccer	Non-elite	108	M	15 to 17	22		X			X	X	
Ambrozy et al. (2021) [25]	Kickboxing	Elite	20	M	25 ± 3	10	X	X					X
Asimakidis et al. (2022) [26]	Soccer	Non-elite	29	M	13 ± 0	32		X				X	
Campa et al. (2021) [27]	Soccer	Elite	15	M	30 ± 4	9	X	X	X		X		X
Cohen et al. (2020) [28]	Soccer	Elite	16	M	24 ± 4	15		X				X	
Dauty et al. (2021) [29]	Soccer	Elite	19	M	14	9							X
Fikenzer et al. (2021) [9]	Handball	Elite	10	M	27 ± 3	44							X
Font et al. (2021) [30]	Handball	Elite	11	M	26 ± 4	16						X	
Freire et al. (2020) [43]	Soccer	Elite	20	M	26 ± 4	6							X
Grazioli et al. (2020) [10]	Soccer	Elite	23	M	26 ± 6	21		X			X	X	
Junaidi et al. (2021) [46]	Combat Sports	Non-elite	100	M	21 ± 2	37		X			X		
Kalinowski et al. (2021) [31]	Soccer	Elite	24	M	15 ± 1	18							X **
Korkmaz et al. (2020) [22]	Soccer	Non-elite	14	M	22 ± 3	12	X	X	X	X	X		
Kosova et al. (2021) [33]	Fencing	Non-elite	15	M&F	16 ± 2	31	X	X					
Leo et al. (2021) [34]	Cycling	Elite	12	M	21 ± 1	30	X	X					X
Luna et al. (2021) [35]	Basketball	Elite	9	M	17 ± 1	16	X	X					
My Giulia et al. (2022) [36]	Soccer	Elite	17	M	22 to 35	51	X	X	X		X	X *	
Paravlic et al. (2022) [37]	Soccer	Elite	32	M	25 ± 5	8	X	X				X	
Parpa and Michaelides (2021) [38]	Soccer	Elite	19	M	28 ± 6	7		X			X	X	X
Pedersen et al. (2021) [39]	Soccer	Elite	13	F	19 ± 2	12						X	
Pucsok et al. (2021) [40]	Soccer	Elite	11	M	17 ± 1	13						X	
Scoz et al. (2022) [41]	Soccer	Elite	26	M	26 ± 5	6	X	X					
Segalés-Gill et al. (2021) [42]	Soccer	Elite	26	M	25 ± 5	11		X					X
Silva et al. (2022) [13]	Badminton	Elite	23	M&F	19 ± 3	48		X	X	X			X
Spyrou et al. (2021) [8]	Indoor soccer	Elite	10	M	26 ± 3	10		X	X		X	X	
Valenzuela et al. (2021) [45]	Badminton	Elite	7	M&F	21 ± 3	22						X	
Villaseca-Vicuña et al. (2022) [44]	Soccer	Elite	19	F	27 ± 4	22		X	X		X	X	
Yasuda et al. (2021) [47]	Fencing	Elite	21	M&F	26 ± 5	36	X	X	X	X	X		

Caption: N = number of subjects; M = Male; F = Female; M&F = Male and female; Weeks = time between first and second assessments in weeks; Fat % = fat percentage; CMJ = Countermovement jump; Combat Sports = Karate, Taekwondo, and Pencak Silat (Indonesian martial arts). * = study not reported for this variable data in mean and standard deviation. ** = data of $\overset{˙}{V}$O2peak reported in meters. X = Variable reported in the current systematic review.

**Table 3:** Meta-regression of moderators for the change in body weight or CMJ variables during COVID-19 lockdown in athletes.

	Moderator	Number of Studies	β Coefficient	95% CI		*p*-Value
Body weight	Age (years)	15	−0.023	−0.027	−0.020	<0.001
	Follow-up time (weeks)	17	−0.006	−0.005	−0.008	<0.001
	Level (Elite or Non-elite)	17	−0.169	−0.200	−0.128	<0.001
CMJ	Age (years)	7	−0.008	−0.009	−0.006	<0.001
	Follow-up time (weeks)	7	−0.065	−0.074	−0.057	<0.001
	Level (Elite or Non-elite)	7	0.076	0.017	0.135	0.012

CI = confidence interval; CMJ = Countermovement Jump.

## Data Availability

The data that support the findings of this study are available from the corresponding author (F.E.R.) upon reasonable request.

## Supplementary Materials

The following supporting information can be downloaded at: https://www.mdpi.com/article/10.3390/healthcare11162319/s1. Supplementary data has data of pre- and post-confinement physical fitness outcomes, and the following topics and data were extracted from each study: title, author, year, study design, participant characteristics, performance or body composition measurement, evaluation protocol and instrument, follow-up time, and training protocol during confinement (Table S1).
