# Supplementary material for: Impact of the COVID-19 Lockdown on the Body Composition and Physical Performance of Athletes: A Systematic Review with Meta-Analysis and Meta-Regression
Source: Healthcare (Basel). 2023 Aug 17;11(16):2319. doi: 10.3390/healthcare11162319 (PMC10454760; doi:10.3390/healthcare11162319)
Supplement: Supplementary file 1 [file healthcare-11-02319-s001.zip › healthcare-2533374-supplementary.pdf]

Supplementary Material

Impact of the COVID-19 Lockdown on the Body Composition and Physical Performance of Athletes: A Systematic Review with Meta-Analysis and Meta-Regression

Bruno Viana Rosa, Alberto Jimenez Maldonado, Ayrton Oliveira de Araújo, Lucas Melo Neves, and Fabricio Eduardo Rossi

S*upplementary* table S1: Training protocol during confinement.

Author, year	Study design	Protocol during follow-up
Aguilar et al. (2021) [23]	Las mediciones fueron realizadas enfebrero de 2020 y justo después del confinamiento en el mes de mayo de 2020, momento en que dio comienzo de nuevo la competición de la Serie A del Calcio Italiano.	Players reported a series of physical exercises recommended to them: 1) Plank 30 s, lateral both sides and bench press. 2) Planks 30 s lateral with activation of Mm. glutes and abductors of both limbs. 3) Activation of Mm. Alternate glutes with sit-ups. 4) Squash on the wall 1 min with 10 kg - Contraction of the gluteus medius muscle with sprint toe support and raised heel of one foot. 5) Isometric background 30 s with instability - Stability of the gluteus medius supporting one foot and touching cones, alternating the leg. 6) Lateral Squash 30 s slow with motion control - Combining Lateral Squash 30 s with change of speed. 7) 5s background combined with speed and rotation change. In addition, players performed unscheduled aerobic exercises, both on a cycle ergometer and on a treadmill, depending on the availability and accessibility of each player in home confinement.
Alvurd et al. (2022) [24]	Performance and body composition were evaluated in soccer players 38 days before isolation caused by Covid19 and 12 days after isolation	During the first 48 days of lockdown, players performed a standardized 40 min home-based training program consisting of body-weight resistance exercises. Players trained individually 3 times a week at home and were monitored online under the supervision of group coaches. During the other 68 days of isolation, the players did not carry out any organized training plan
Ambrozy et al. (2021) [25]	The first measurement was performed before the restrictions on access to sporting facilities were imposed on 13 March 2020. The second measurement was carried out on 21 May 2020, after the end of the lockdown and reopening of sports clubs. Was measured: Aerobic Capacity Test; 50-meter Sprint; Standing Long Jump; 1000-meter Run; Grip Strength; Pull Up; 5 × 10-meter Shuttle Run; Sit-Ups; Forward Bend.	The participants were first or master class athletes according to the Polish Kickboxing Association. The frequency of training sessions before the lockdown was 6 times a week, 2 h each. The coaching recommendations for the period of restrictions caused by the pandemic were the same. The athletes were measured for body mass, body height, and physical fitness. Body mass was measured using a Tanita BC-601 body composition monitor (Tanita, Tokyo, Japan), the height was measured using a SECA 2017 body height meter (Seca, Hamburg, Deutschland), and physical fitness was evaluated based on the following ICSPFT tests [26]:

Asimakidis et al. (2022) [26]	This study aimed to investigate the effects of the lockdown implementation, due to the COVID-19 pandemic, on the performance of SSC actions in young soccer players. The suspension of the training process lasted approximately 32 weeks (128 days), and during this period, only a small amount of physical activity was possible. Moreover, no structured online training guidance was delivered. The participants were measured twice: the first testing period was just before the beginning of the first national lockdown in Greece (TP1), whereas the second testing period took place one week after the youth soccer academies resumed their operation (TP2). Change-of-direction ability of both legs (505 change of direction test), linear speed (10 m and 20 m sprint tests) and vertical jumping ability (countermovement jump—CMJ), were the neuromuscular performance indicators evaluated. Furthermore, the maturity level, expressed as years from PHV, was calculated for each athlete in both testing periods. Subjects were familiarized with the testing procedures prior to the first measurement.	During thie 32 weeks, only a small amount of physical activity was possible. Moreover, no structured online training guidance was delivered.
Campa et al. (2021) [27]	The study carried out an assessment of body composition in four moments, in 2020 in the pre (end of February) and post (end of May) lockdown and in 2021 on the same dates.	During COVID-19 confinement, 4 sessions of 45 minutes per week were requested to work strength of the upper and lower limbs, and to preserve aerobic abilities: cardio-training and HIIT (high intensity interval training)
Cohen et al. (2020) [28]	We compared data for 16 first-team players of América de Cali (current Colombian Premier League champions) who had performed CMJ tests 1) just before COVID-19 league suspension in February 2020 and 2) on their first day of return from quarantine at the end of June 2020, following 15 weeks of isolated training	Home ‘team’ workouts (circuit training) via Zoom (5/wk).
Dauty et al. (2021) [29]	Study verified the effect of the lockdown by Covid-19,. on cardiorespiratory capacity and psychological factors, in adolescents who performed online training during the pandemic. Assessments were carried out pre and post lockdown	During COVID-19 confinement, 4 sessions of 45 minutes per week were requested to work strength of the upper and lower limbs, and to preserve aerobic abilities: cardio-training and HIIT (high intensity interval training)

Fikenzer et al. (2021) [9]	Study collected data from 3 points on the timeline, July 2019 (P1), January and post-lockdown in May 2020 (P2). The results of shuttle run tests and incremental test in P1 and P2 were compared	An unmonitored training program was performed during the 8 weeks of blockade. The training plan consisted of three training days, day 1 included stabilization training and endurance I (high-intensity interval training short) exercises; day 2 included strength training legs and endurance II (continuous training) exercises; day 3 included strength training upper body and endurance III (high-intensity interval training long) exercise
Font et al. (2021) [30]	A retrospective design was used to compare the change in submaximal shuttle run test and jump test performance. A 9-week home-based training programme was followed during lockdown. Pre-test measurements were assessed before the pandemic on 29 January 2020 and ended on 18 May 2020.	Players performed an average of 27 strength training sessions, including both individual sessions and online group sessions. Regarding endurance training, players performed an average of 19 sessions. In the first four weeks, players performed individual strengthbased high-intensity interval training (HIIT) circuits, and from the fifth week onwards they were prescribed general aerobic fitness training sessions based on continuous and progressive exercises.
Freire et al. (2020) [43]	Verified the effects of quarantine during a severe viral outbreak on cardiovascular performance and associated with the Yo-Yo test in a sample of professional soccer players.	Three times a week for 30 min per session of aerobic training between ~65 and~75% of maximal heart rate.
Grazioli et al. (2020) [10]	The players were tested after the traditional off-season period (24 days; November 23, 2019, to December 17, 2019), just before the start of the pre-season, and after the COVID-19 quarantine (63 days; March 16, 2020, to May 18, 2020). Study assessed body composition, jump and sprint performance, hamstring eccentric strength, and intermittent cardiorespiratory fitness	Players were instructed to perform home-based workouts using only the body mass as resistance during quarantine (i.e., warm-up of knee/hip mobility exercises and 3 sets of 10–15 repetitions of vertical jumps, jumping lunges, lateral squats, isometric hip thrust, isometric squat, plantar flexion, Nordic hamstring exercise, unilateral stiff, inverse Nordic exercise, horizontal jumps, and 30–60 seconds of skip and core exercises).

Junaidi et al. (2021) [46]	In 2020, DKI Jakarta, the capital of Indonesia implemented a large-scale social restrictions (LSCR) policy from 14 April to 21 December. During this period, all activities in the region were heavily restricted, including sporting activities. All regional athletes in DKI Jakarta were mandated to train and exercise independently at home, and sent the report training to coach by online. In consideration of this restriction, the results of this study were categorized into two parts: body mass measurement before the large-scale social restrictions in DKI Jakarta (Pre-LSCR) and body mass measurement during the large-scale social restrictions in DKI Jakarta (During-LSCR). Pre-LSCR was conducted on 10–12 April 2020 and During-LSCR was conducted on 28 December 2020.Pre-LSCR measurement was conducted directly at the sports hall of KONI DKI Jakarta. It was a collection of preliminary data in preparation for all athletes to self-quarantine.	All regional athletes in DKI Jakarta were mandated to train and exercise independently at home, and sent the report training to coach by online.
Kalinowski et al. (2021) [31]	Over the period of one year, players took part in the Beep Test six times. The mean levels of endurance were compared in the analysis in six periods of measurement (T1–T6). T 1—January 2020—Start of tests; T 2—March 2020—1 week before the lockdown; T 3—May 2020—1 week after the lockdown; T 4—June 2020—4 weeks after the lockdown; T 5—July 2020—8 weeks after the lockdown; T 6—January 2021—finishing the study after a year-long cycle.	During the eight week quarantine the players participated in training intervention at home (in their own houses or flats). The training sessions took place online using the ZOOM platform. All exercises were performed with low intensity, using household equipment, such as 1–2 kg dumbbells or a bottle of the same weight, exercise mat, massage roller, chair, or skipping rope. The intensity of the exercises was monitored continuously and adjusted to the self-perceived level of effort, which was analysed by the coaching staff after each training session using the Borg rating of perceived exertion scale of 1–20. The Borg scale (RPE) is a method used to monitor exercise intensity in a subjective way. An RPE of 1–9 indicates low intensity, while an RPE of 10–13 and >14 indicates high intensity. RPE is a universal tool, irrespective of locomotor mode and variations in terrain and environmental conditions. Video analysis was used for the assessment of the quality of exercises in real time.
Korkmaz et al. (2020) [22]	Performance, flexibility and body composition variables of soccer players were collected pre and post isolation during the Covid-19 pandemic.	Light and irregular exercises with body weight.

Kosova et al. (2021) [33]	The pre-tests were conducted in September 2019 (before the COVID-19 pandemic process) in the middle of the competition season. From the date of the measurements, fencers continued their weekly routine training (week/4 days). Then, with the emergence of the COVID-19–induced pandemic process on March 11th, 2020, they had to take a break from their regular training. The tests of the fencers, who started their training again on 19 October 2020, were performed on October 26th, 2020, after a 31-week detraining period.	N/A
Leo et al. (2021) [34]	The whole intervention involved 90 days, which were equally divided into 30 days pre, during, and post COVID-19 restrictions.	The accumulated training hours, distance covered, and training frequency per week were recorded for the respective periods, mentioned above. All athletes uploaded their training data to an online training platform (Trainingpeaks, Trainingpeaks LLC, Winchester Cir, MA, USA) [14]. Weekly training hours, distance covered, and training frequency were collected and further processed, analyzed, and checked for data spikes in Microsoft Excel (Excel, Microsoft Corporation, Redmond, WA, USA). Intensity ratios, including distance per hour (km·hour−1) and distance per session frequency (km·session−1), were calculated.
Luna et al. (2021) [35]	This non-experimental, quantitative and longitudinal study (pre-post) was carried out for 4 months. Four days prior to the declaration of total cessation of all activity, an evaluation was carried out through a battery of physical tests. During the validity of this state of "alarm", the participants followed a remotely supervised training plan, and 12 weeks later, in accordance with the end of the mandatory quarantine, they underwent a second evaluation with the same battery of tests. evidence.	During the 12 weeks that the confinement lasted, four night sessions were held per week (Monday, Tuesday, Thursday and Friday), with 50 effective minutes divided into activation blocks, central part and cooling down. The sessions consisted of dynamic and static stretching, resistance training, endurance and specific training with jumps, accelerations and decelerations.
My Giulia et al. (2022) [36]	T0 was scheduled before the start of the preparatory period (week 0; middle July); T1 was about at the beginning of the championship (week 14; October); T2 was programmed in the middle of the championship (week 25; January); T3 was in March (week 35); and T4 was at the end of the season (week 51; June).	4 or 5 aerobic sessions performed at home with fixed devices (treadmill or bike) or with bodyweight + 2 or 3 strength training sessions using body weight and small weights + 1 or 2 running sessions,
Paravlic et al. (2022) [37]	To address this issue, elite soccer players from the Slovenian Premier League were	Home-based training programs

	assessed before (PRE) and 11 days following (POST) the COVID-19 lockdown period. The PRE tests were performed in the framework of the national project (L5-8245), where the last measurements took place between 6 and 20 June 2019, and POST tests were additionally organized.	
Parpa and Michaelides (2021) [38]	This observational study compared the same players' anthropometric and physical fitness obtained right after the 5-week transition period to those obtained after the 7-week COVID-19 lockdown. All the participants had medical clearance and a negative COVID-19 polymerase chain reaction (PCR) test within 48–72 hours before the testing. Fitness testing was conducted on two different testing days to avoid potential fatigue from subsequent testing and give enough time to sanitize the room surfaces and equipment between the players.	During the 7-week lockdown the players were provided with an individualized training protocol similar to the one they were instructed to follow during the 5-week transition period. Microcycles (each week) were identical in structure and included three strength/power training sessions per week and four cardiovascular sessions on alternate days. More specifically a player had to follow a strength training session on Monday, Wednesday, and Friday while cardiovascular sessions were recommended for Tuesday (sprints), Thursday (speed intervals), Saturday (tempo intervals) and Sunday (continues running for 45 min). Each strength training session consisted of a tenminute warm up that included low to moderate intensity aerobic exercises and a five- minute cool down that included stretching exercises.
Pedersen et al. (2021) [39]	longitudinal 12-week observational study with a pretest posttest design. Two female football teams playing at level three in Norway were originally invited to another study, whose main aim was to investigate the association between high-force/power tests and physical performance derived from tracking data during football match play. Since the COVID-19 lockdown was imposed by national authorities 1 week following pretest start, and 1 day prior to pretest the second team (n = 13)	Soccer-specific individual training in strength, endurance, jumps and speed
Pucsok et al. (2021) [40]	We conducted the first measurement at the end of the preparatory period, on 28 February 2020. The second session consisted of 4 weeks of regular training and 9 weeks of individual, home-based activities.	Were prescribed exercises to be done at home. Each training session's duration was 60 min. The participants performed 12 min of a warm-up session, including a mobilization block and dynamic stretching. Finally, a 6 min cool-down (easy jogging and stretching) session was performed. Participants monitored intensity individually by using the modified Rate of Perceived Exertion scale.
Scoz et al. (2022) [41]	This is a retrospective two-arm cohort study based on isokinetic data from Brazilian professional elite-level soccer players evaluated prior to and after the COVID-19 quarantine period. We decided to compare two home training strategies used by each team's athletic trainer during the official	They were divided into two groups and trained strength, endurance and power accompanied by video call or without this monitoring

	lockdown period (6 weeks of quarantine). Also, we compared the results from pre-postquarantine with their isokinetic records pre- and post-interseason period (4 to 6 weeks).	
Segalés-Gill et al. (2021) [42]	Performed stress test evaluation, in ergospirometry, in professional soccer players. Being the first assessment in the 2020 pre-season and the second after 11 weeks of detraining	More than 60% of the players performed at least three training sessions per week; of which 50% did high-intensity running and the other 50% did not do high-intensity aerobic training. Regarding continuous running for at least 20 minutes, 75% of those who did more than three workouts answered yes to this last question. None of them did specific strength training.
Silva et al. (2022) [13]	Athletes were requested to stop their regular training routine in March 2020. Thus, we performed the baseline assessments on 32 athletes in June 2020, after 3 months of COVID-19 social restriction. Then, in February 2021, 28 athletes were randomized into two groups: retrained group = 14 athletes, who stopped their daily training routine for 8 months due to COVID-19 social restriction plus 4 months of retraining; and detrained group = 14 athletes, who stopped their daily training routine for 1 year due to COVID-19 social restriction. After 4 months (June 2021), the athletes were evaluated again, .	During the pandemic, the coaches used video classes on the Google Meet platform, three times a week for about 60 min per day to guide their athletes at home to keep themselves fit and healthy, focusing only on physical capabilities, such as flexibility, aerobic capacity, and agility, and not on technical or tactical training
Spyrou et al. (2021) [8]	This study evaluated futsal players in the pre (March 12) and post (May 13) confinement by Covid-19. Jumping, sprinting and body composition variables were investigated.	They performed a semi-structured maintenance training program consisting of exercises using only body mass as resistance. Athletes were instructed to perform these exercises 2 to 3 times per week, completing 2 or 3 sets of 6 to 8 (jumps) and 10 to 12 (squats and lunges) repetitions.
Valenzuela et al. (2021) [45]	This study retrospectively analyzed data from badminton athletes provided by their training teams. Data from February to July 2020, including 4 weeks of usual training (baseline), 7 to 10 weeks of isolation, and 6 to 8 weeks of retraining, were analyzed.	~8–12 Training sessions in total per week (60–70 min·session−1). Sessions included HIIT (including badminton-specific tasks when possible) and RT circuits. All athletes had access to elastic bands and light to moderate weights. Only one athlete had access to a cycle ergometer, a hypoxia generator, BFR bands, heavy weights, and inertial systems.
Villaseca-Vicuña et al. (2022) [44]	Assessments of body composition, endurance, strength, and speedpower related performance pre and post blockade caused by Covid-19 were carried out. Assessments took place in the last week of February 2020 and the first week of August. The lockdown ran from March 11, 2020 to July 22, 2020	All participants trained at home under the supervision of the coaching staff (through videoconference, three weekly sessions).
Yasuda et al. (2021) [47]	September 2019 for baseline, and of 21 (12 men, 9 women) who completed the following	N/A

	measurements in June 2020 (POST; immediately after rescinding the emergency state) and September 2020 (POST-4M; 4-months after rescinding the emergency state).	
